# Androgen receptor-binding sites are highly mutated in prostate cancer

**DOI:** 10.1038/s41467-020-14644-y

**Published:** 2020-02-11

**Authors:** Tunç Morova, Daniel R. McNeill, Nada Lallous, Mehmet Gönen, Kush Dalal, David M. Wilson, Attila Gürsoy, Özlem Keskin, Nathan A. Lack

**Affiliations:** 1 School of Medicine, Koç University, Istanbul, 34450 Turkey; 2 Vancouver Prostate Centre, University of British Columbia, Vancouver, V6H 3Z6 BC Canada; 3 Laboratory of Molecular Gerontology, National Institute on Aging, NIH, Baltimore, MD 20892 USA; 4 College of Engineering, Koç University, Istanbul, 34450 Turkey; 5 Hasselt University, Diepenbeek, BE3590 Belgium; 6 Koç University Research centre for Translational Medicine (KUTTAM), Koç University, Istanbul, 34450 Turkey

**Keywords:** Cancer genomics, Prostate cancer, Hormone receptors

## Abstract

Androgen receptor (AR) signalling is essential in nearly all prostate cancers. Any alterations to AR-mediated transcription can have a profound effect on carcinogenesis and tumor growth. While mutations of the AR protein have been extensively studied, little is known about those somatic mutations that occur at the non-coding regions where AR binds DNA. Using clinical whole genome sequencing, we show that AR binding sites have a dramatically increased rate of mutations that is greater than any other transcription factor and specific to only prostate cancer. Demonstrating this may be common to lineage-specific transcription factors, estrogen receptor binding sites were also found to have elevated rate of mutations in breast cancer. We provide evidence that these mutations at AR binding sites, and likely other related transcription factors, are caused by faulty repair of abasic sites. Overall, this work demonstrates that non-coding AR binding sites are frequently mutated in prostate cancer and can impact enhancer activity.

## Abstract (web summary)

Androgen receptor (AR) mediated transcription is critical to prostate tumorigenesis and development. Here, utilising clinical whole genome sequencing data, the authors show that the non-coding AR binding sites on DNA are frequently mutated in prostate cancer potentially due to faulty base excision repair mechanisms

## Introduction

Cancer arises through the sequential accumulation of mutations that induce neoplastic transformation and uncontrolled proliferation. Each mutation can provide remarkable insight into the history of the cancer as different mutation types arise from different events^1^. Somatic mutations do not occur in a normal distribution across the genome and are affected by several variables including GC content, replication time, distance to telomere, and chromatin compaction^2–4^. Recent studies have demonstrated that transcription factor (TF) binding to DNA can also correlate with a higher rate of mutations^5,6^. Elegant work combining XR-seq from UV-treated skin fibroblast cells and large-scale whole-genome sequencing (WGS) demonstrated that in skin cancer, TF-binding impairs nucleotide excision repair machinery (NER)^7^. By physically preventing the access of repair enzymes, TF binding causes a higher rate of UV-mediated mutations in skin cancer. However, it is unlikely that only NER is affected by TF binding given the diversity of endogenous mutations observed in different cancer types.

Prostate cancer (PCa) is an extremely common disease that affects an estimated one out of every seven North American men in their lifetime. At all stages of PCa development, androgen receptor (AR)-mediated transcription is critical to the growth of the tumor. Following activation, the AR translocates from the cytoplasm to the nucleus where it interacts with pioneer factors such as FOXA1 before binding to chromatin. The vast majority of AR-binding sites (ARBS) are located in intronic or intergenic regions^8,9^. Many of the ARBS contain an androgen response elements (AREs) that consists of a 15-bp palindromic sequence containing two hexameric 5′-AGAACA-3′ half sites arranged as an inverted repeat with a 3 bp spacer^10,11^. Once bound to DNA, the AR recruits various co-activators that eventually initiate transcription of pro-mitotic genes. Several factors have been demonstrated to affect AR-mediated transcription such as epigenetic modifications, pioneer factors, and chromatin accessibility^9,12^. Demonstrating the importance of these co-activators and pioneer factors, FOXA1, HOXB13, GATA2, and KDM1A have been shown to be critical for AR signaling and are required for the growth of PCa cell lines^13–16^. In addition to initiating PCa growth, there is also evidence that AR signaling is associated with DNA damage. Goodwin et al. demonstrated a feedback loop whereby DNA repair genes activate the AR upon DNA damage and subsequently promote DNA repair^17^. Further, AR has itself been shown to induce double stranded breaks (DSB) via topoisomerase IIb (TOP2B)^18^. Specifically, AR recruits TOP2B to introduce DSB that relax torsional stress and allow transcription. These DSB are not typically recombinogenic and can be repaired by DNA repair mechanism. However, additional genotoxic stress can prevent repair and increase the rate of DSBs by activating induced cytidine deaminases or LINE-1 repeat-encoded ORF2 endonucleases, thereby leading to structural variations, including the common TMPRSS2:ERG fusion^19^.

There has been extensive research to identify protein-coding “driver” mutations in both primary and castrate-resistant PCa^20,21^. From these large studies, numerous deletions (PTEN, CADM2), structural variants (ETS fusions), and single-nucleotide variations (FOXA1, SPOP) have been identified as potential driver mutations in primary PCa. However, until recently the impact of non-coding mutations has been poorly understood. This is changing as their importance is increasingly becoming more evident in other cancer types. One of the first non-coding driver mutations identified was found at the promoter of telomerase reverse transcriptase (TERT)^22,23^. This mutation caused increased *TERT* expression and repair of shortened telomeres^24^. In PCa, recent work by several laboratories demonstrated that duplication of an AR enhancer acts as a common driver of castrate-resistant PCa^25–27^. Given their potential role in modifying the transcriptional landscape of PCa, a better understanding of non-coding variants is critical to identifying novel driver mutations.

Although AR has been previously shown to induce DNA damage in vitro, the relatively low frequency of somatic mutations in primary PCa (~ 1 SNV per Mb) has prevented the study of TF-mediated DNA damage in clinical samples. Therefore, using large-scale WGS data we investigated how TF binding affects somatic mutations in PCa^28^. Interestingly, we found that AR binding causes a high level of somatic mutations at ARBS and that the mutations are likely caused by impaired DNA repair.

## Results

### ARBS have a markedly higher rate of mutations in PCa

**Fig. 1:**
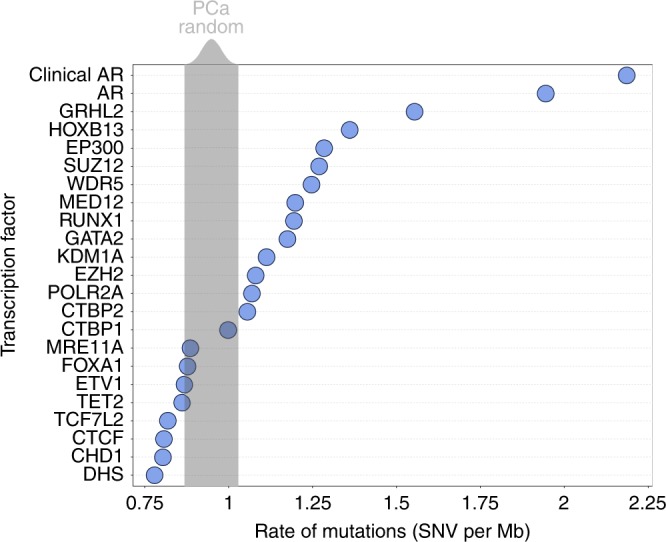
ARBS sites are the most heavily mutated TF-binding sites. The rate of mutations (SNV per Mb) at individual TF-binding sites (*n* = 22) and DHS regions were compared with randomized chromosomal regions (1000 iterations, gray). All TF-binding data were generated from a secondary cell line except “Clinical AR” that are high-confidence ARBS from patients PCa samples.

To investigate the impact of TF binding on non-coding somatic mutations, we initially quantified the mutational density at binding sites using WGS of primary PCa (*n* = 196) from the Pan Cancer Analysis of Whole Genome (PCAWG). TF-binding sites were obtained from ChIPseq of a prostate cancer cell line (LNCaP) and clinical samples when available. DNA hypersensitive sites (DHS) were included as a negative control, as DHS were shown to have a lower rate of somatic mutations owing to increased access of DNA repair machinery^29^. When we compared the mutational rate at TF-binding sites to randomly shuffled regions in PCa, many TF-binding sites including HOXB13, EP300, SUZ12 were found to have a statistically higher rate of mutations (false discovery rate = 0; Fig. 1). As expected, DHS had less mutations than any TF or random regions. Contrasting earlier work in both colorectal cancer^5^ and melanoma^7^, CTCF-binding sites did not have an increased rate of mutations as compared to either random regions or regions nearby the TF-binding site (Supplementary Fig. 1A). However, of all the TF’s characterized, ARBS were found to have the highest rate of somatic mutations. Confirming that this was not a cell line-specific artifact, we observed an even greater mutational rate at ARBS from clinical ChIPseq (Fig. 1). A similar trend was observed with indels at ARBS in PCa, though not as dramatic owing to the low numbers of indels obtained by consensus mutation calling (Supplementary Fig. 1B). The increase in ARBS mutations is not likely owing to epigenetic modifications as ARBS had greater than twice the mutation rate of regions with H3K27Ac, H3K4me3, H3K4me1, or H3K36me3 marks.

**Fig. 2:**
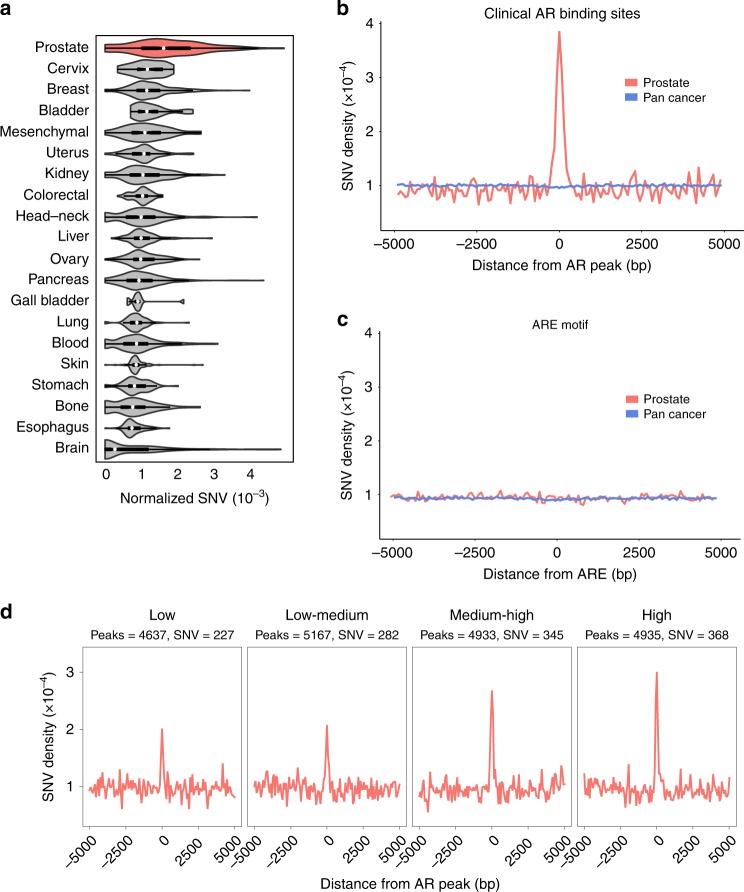
ARBS have an increased rate of mutations only in PCa. **a** PCa (red) has the highest normalized SNV rate at clinical ARBS of all cancer types. **b** The mutational density ±5 kb at clinical ARBS was markedly increased in PCa (red) but not in all other cancers (blue). **c** A similar analysis was done at regions in the genome that had the canonical ARE motif but no AR binding. No increase in mutational rate was seen in either PCa (red) or other cancers (blue). **d** AR ChIPseq peaks were divided into quartiles based on peak height (low/low–medium/medium–high/high). A clear correlation was observed between peak height and increased SNVs at ARBS.

AR provides an ideal model to study TF-mediated mutations as this nuclear receptor is critical to the growth of nearly all PCa tumors, but is not active or required in other cancers. Thus, the same ARBS chromosomal locations should not have increased mutations in other cancers if the observed results are due to AR binding rather than regional DNA instability. When we calculated the rate of mutations at ARBS from WGS of over 20 different cancer types (*n* = 2576), the rate of SNV mutations at ARBS was greater in PCa than either all other cancers (Wilcox *t* test; *p* *<* 2 × 10^−16^) or any individual cancer (Fig. 2a). Importantly, no cancer other than PCa had a significant increase in SNVs at ARBS (Supplementary Fig. 2). An increase in mutations at ARBS was clearly observed in PCa, but not other cancers, with a enrichment ~ ± 375 bp from the maximal AR peak (Fig. 2b). This was not owing to nucleotide composition, as those regions that have an ARE motif but no bound AR did not have an increase in SNVs or indels (Fig. 2c). Providing further confidence that these mutations occur owing to AR occupancy, we observed a clear correlation between SNV density and ChIPseq peak height (Fig. 2c). Overall these results demonstrate that AR binding correlates with an increase in somatic mutations. To determine whether a similar increase in mutations was observed with other lineage-specific TFs, we quantified the rate of SNVs at estrogen receptor-binding sites (ERBS) in breast cancer (Supplementary Fig. 3). Similar to what we observed at ARBS in PCa, breast cancer had the highest rate of mutations at ERBS. Although not the goal of this work, it does suggest that TF-binding site mutations are cell-of-origin specific. We then looked to determine whether the ARBS mutations occurred at those regions with specific epigenetic modifications or TF binding co-occupancy. This was based on previous literature that demonstrated that the cellular epigenetic state could dramatically alter the mutational rate^2^. However, no relationship could be observed between ARBS mutations and specific histone marks or TF co-occupancy (Supplementary Fig. 4). As we do not have binding information for all possible histone marks or TF there may yet be an undiscovered correlation. However, our current data suggest that ARBS mutations do not correlate with specific epigenetic modification or proteins and are solely due to AR binding.

### AR-mediated SNV mutations induce purine transversions

**Fig. 3:**
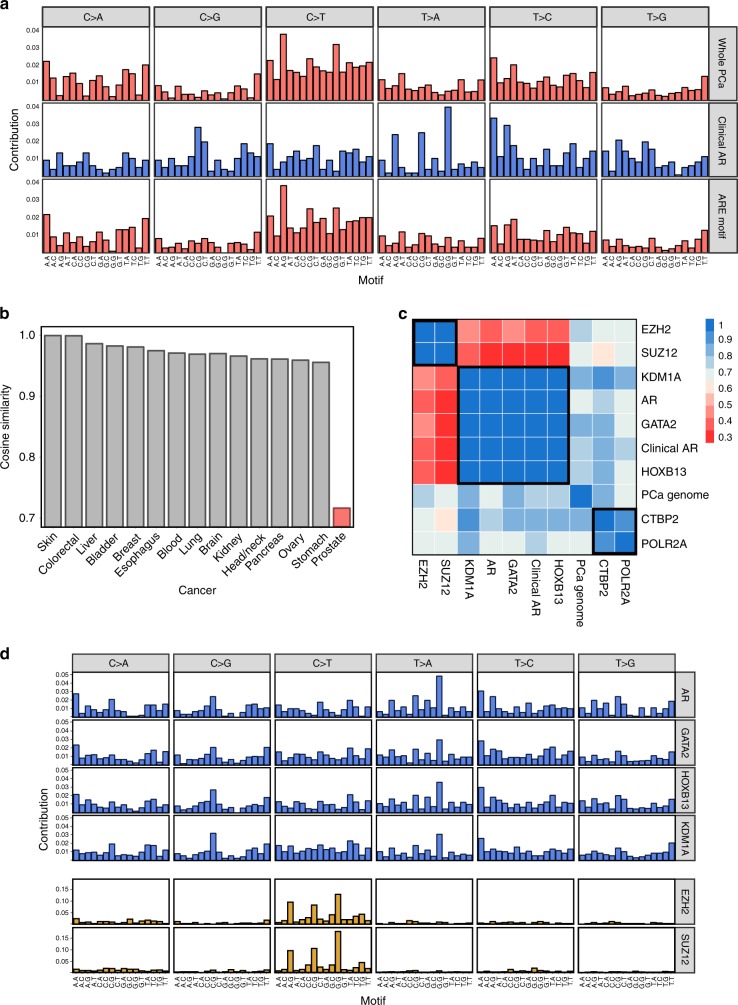
ARBS have a different mutation signature. **a** The type of mutations at clinical ARBS were compared with all those found in the whole PCa genome or those regions that contain an ARE motif but no AR. **b** The mutational signature at ARBS chromosomal regions was compared with the remainder of the genome in multiple cancer types. Only PCa was found to have an altered mutational signature at ARBS. **c** The mutational signature of each TFs-binding site was compared and three clusters of mutation types were observed at SUZ12/EZH2 [group 1], KDM1A/AR/GATA2/HOXB13 [group 2] and CTBP2/POLR2A [group 3] **d**. Detailed analysis of the group 1 and group 2 are shown.

To better understand the cause of these mutations, we then determined the mutational signature at ARBS. Although these binding sites only represent a portion of the total genome (~ 100 Kb), we proposed that the mutational signature of a large region should be roughly the same as the whole genome if there is a sufficient number of mutations. Supporting this, we found that random regions with a similar size or nucleotide composition to ARBS almost always had a near identical mutational signature to the PCa genome (Supplementary Fig. 5A). Further, the number of SNVs observed at ARBS are well over the previously calculated minimum threshold to decipher a mutation signature with > 95% accuracy^30^. Interestingly, when we looked at the mutations at ARBS in PCa we found a dramatically different mutational profile than the remainder of the cancer genome (Fig. 3a). Specifically, there was an increase in TpG- > ApG and CpG- > GpG purine transversions. These infrequent mutations occur at a much lower rate in the remainder of the PCa genome. Demonstrating that this was not due to the nucleotide composition, those regions of the chromosome that have an ARE motif but no AR binding did not have the same type of mutations (Fig. 3a). When we shuffled chromosomal locations to match the nucleotide composition of ARBS and recalculated the mutational signature, no random regions were found to have mutational signatures comparably enriched for TpG - > ApG transversions (Supplementary Fig. 5B). We observed no difference in either the rate or type of mutation if the ARBS had a canonical ARE (Wilcoxon signed rank test *p* = 0.97*;* Supplementary Fig. 6). This suggests that it is protein occupancy that correlates with SNVs. Finally, to test if the mutations were simply owing to the specific chromosomal locations where AR binds we compared the mutational signatures at ARBS in all cancer types (Fig. 3b). Only PCa was found to have a different mutation type at ARBS. All other cancers, which do not express or require AR, had the same signature at both the ARBS and whole genome. This demonstrates that the observed ARBS mutational signature was not caused by differences in nucleotide composition or chromosomal characteristics and is owing to AR occupancy.

Having observed an AR-specific mutational signature, we tested if other TF-binding sites had similar types of mutations. We speculated that if these mutations were directly caused by AR binding, only ARBS would have this signature. We therefore analyzed all TFs that had both an increased rate of mutations (Fig. 1) and a total number of mutations that was greater than the previously published theoretical threshold^30^. When the TF mutation signatures were compared, we found three distinct signature types (Fig. 3c). In the first, KDM1A, HOXB13, and GATA2 were found to have a very similar mutational signature to AR (Fig. 3d). This correlation was not due to co-occupancy of the binding sites as a similar result was obtained even after removing regions that overlap with the AR (Supplementary Fig. 7A+B). Further, it was not owing to the nucleotide composition of these regions as those site with AR, GATA2, or HOXB13 motifs but no protein (motif alone) did not have either an increased rate of mutations or a change in the mutation type (Supplementary Fig. 7A+B). The TpG- > ApG and CpG- > GpG purine transversions were only observed in PCa and not seen in other cancer types (Supplementary Fig. 7C). In the second signature members of the polycomb repressive complex 2 (PRC2) including SUZ12 and EZH2 had a strikingly distinct signature containing almost exclusively CpG- > TpG transitions. This was not owing to simple overlap of the binding sites between these TFs (Supplementary Fig. 8A). However, these mutation types were not only seen in PCa. We also observed a similar mutational signature at SUZ12/EZH2-binding sites in several other cancer types (Supplementary Fig. 8B). Finally, the remaining TFs including POLR2A and CTBP1 had a complicated mutational signature that was much closer to the whole genome than the other TFs. Importantly, the observed mutational signatures were not solely due to nucleotide composition as POLR2A, which has a similar GC content to SUZ12 and EZH2, had a very different mutational signature (Supplementary Fig. 9).

**Fig. 4:**
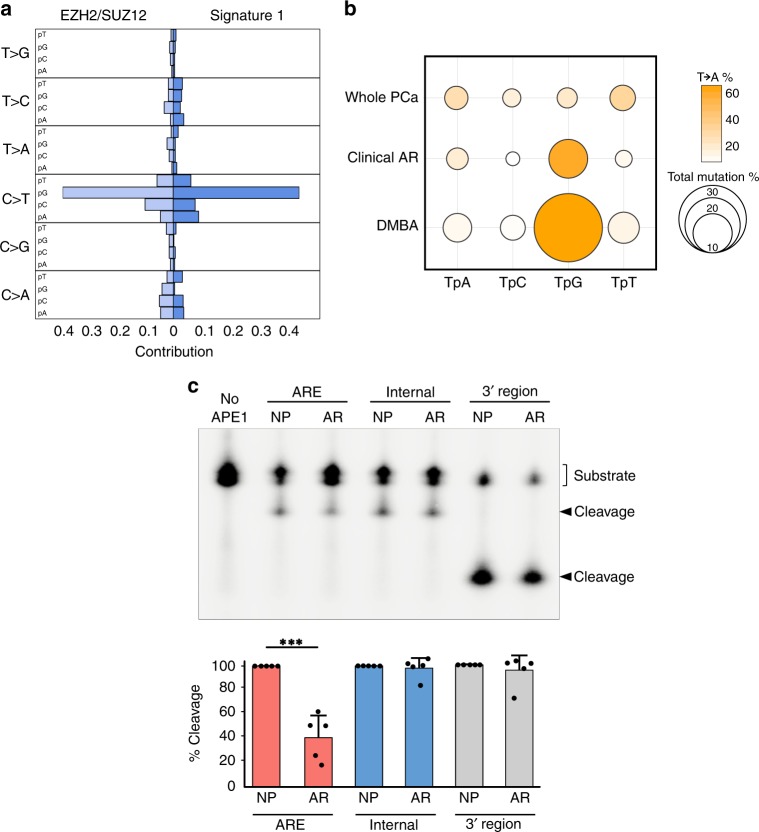
Mutation at PRC2-binding sites and ARBS are similar to previously published work. **a** EZH12 and SUZ12 mutations were concatenated into a PRC2 signature and compared with COSMIC signature 1. **b** The frequency and nucleotide composition of T- > A transversions were compared between the whole PCa, clinical ARBS, and DMBA-treated mice. The majority of the T- > A mutation in both ARBS- and DMBA-treated mice were found to occur at TpG dinucleotides. **c** AR-DBD when bound to abasic DNA reduces APE1 cleavage. Radiolabled duplex DNA with an abasic site at the ARE (ARE), adjacent to the binding site (Internal) and removed from binding site (3’ region) were incubated with either no protein (NP) or AR-DBD (AR) and APE1. Cleavage of the product was quantified and then normalized to the no AR-DBD (*n* = 5, mean ± SD, ***p < 0.0001).

To identify the potential etiological factor of the TF-mediated mutations we compared our results to previously published mutational signatures^1^. Demonstrating the utility of this method, there was a striking similarity between SUZ12/EZH2-binding sites and a previously published COSMIC mutational signature (*Signature 1*; Fig. 4a). This well-characterized signature has been reported in numerous cancer types and is caused by spontaneous deamination of 5-methylcytosine. Supporting this hypothesis, almost all C- > T mutations in SUZ12/EZH2-binding sites were found to occur at CpG sites (Supplementary Fig. 8C). Further, when we looked at genome-wide bisulfite sequencing, SUZ12/EZH2-binding sites had one of the highest levels of DNA methylation (Supplementary Fig. 8D). Having shown the effectiveness of this approach, we then investigated the mutation signature at ARBS and KDM1A/HOXB13/GATA2-binding sites. However, the ARBS mutations were very different than the published COSMIC mutational signatures. Only the signature caused by aristolochic acid had an increased frequency of TpG- > ApG mutations (Fig. 3a). However, the aristolochic acid mutation signature (*Signature 22*) was excluded as it also had many additional T- > A mutations that were not observed at ARBS. Interestingly, outside of the COSMIC database, the uncommon TpG- > ApG purine transversions have been previously shown to be caused by faulty repair of abasic sites. This was demonstrated with the carcinogen dimethylbenzantracene (DMBA), which forms a chemical adduct via one-electron oxidation that depurinates deoxyadenosine nucleotides^31–34^. This massive increase in depurination overwhelms the base excision repair (BER) machinery, causing numerous TpG- > ApG mutations owing to the so-called “A-rule” whereby adenine substitutions are most likely to occur at unrepaired abasic sites^35^. Although it is not likely the prostate will be exposed to these polycyclic aromatic hydrocarbons we proposed that the same faulty BER could potentially cause the observed ARBS mutational signature. Specifically, the presence of AR or other bound TFs could prevent the repair of spontaneously depurinated abasic sites. Such endogenous DNA lesions are extremely common and if not successfully repaired would cause the observed purine transversion. In support of this model, the ARBS mutational signature correlates well to that observed in DMBA-treated animals^33^ (Fig. 4b). To provide orthogonal evidence of this proposed mechanism, we tested the effect of AR binding on repair of abasic sites in vitro (Fig. 4c). In this, we quantified the rate of DNA cleavage by the major human apurinic/apyrimidinic endonuclease (APE1) at an abasic site located at several different locations following binding of AR-DBD to the DNA. APE1 cleavage is an essential step in BER and is required for successful repair of an abasic site^36^. In agreement with our clinical data, we observed that the presence of the AR-DBD protein significantly impacted the cleavage efficiency of APE1 at the abasic site (Fig. 4c). This inhibition was substrate-specific, with APE1 activity only being blocked by the AR-DBD when the abasic lesion was located in the ARBS. Overall, our data support a model whereby AR-DNA complex formation can interfere with the efficient repair of abasic sites, seeding potential mutagenic outcomes during chromosome duplication.

**Fig. 5:**
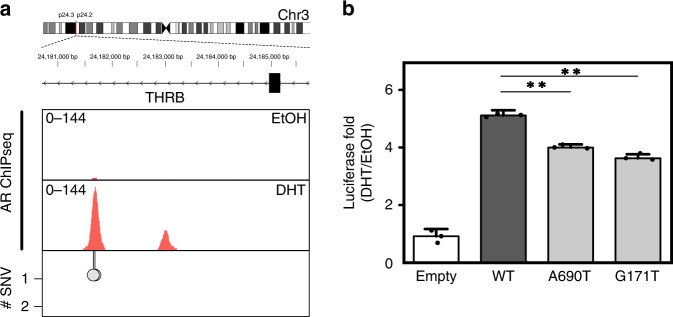
SNVs at ARBS can impact enhancer activity. **a** SNVs were found from two separate patients at an ARBS in the intronic region of thyroid hormone receptor beta (THRB). **b** Clinical SNVs mutations (dark gray) significantly impaired luciferase activity as compared with the wild-type enhancer (light gray) (*n* = 3, mean ± SD, ** *p* < 0.001).

The frequency of ARBS mutations and importance of AR signaling in PCa cancer progression suggest that these somatic mutations could potentially alter gene transcription. Interestingly, several ARBS were identified that had higher than expected mutation frequencies (Wilcoxon rank sum test; *p* *=* 0.015). To explore the effect of these SNVs, we tested the impact of a commonly observed somatic mutation on AR-mediated enhancer activity (Fig. 5). We found that either of the observed SNVs at the THRB ARBS could significantly decrease the AR enhancer. These results demonstrate SNVs in ARBS can alter the AR enhancer activity.

## Discussion

Cancer is largely caused by the accumulation of mutations. Through these, we can begin to understand the molecular underpinnings of the malignant state. However, somatic mutations are not evenly distributed through the genome and are affected by numerous variables. There is emerging evidence that the rate of somatic mutations is higher at TF-binding sites^5–7^. To date, this has been demonstrated only in cancer types that have very high rates of mutations (~ 100 SNV per MB) in order to provide sufficient statistical power. To determine whether this phenomenon occurred in other cancers that have a much lower rate of mutations, such as PCa, we used the recently released WGS from the PCAWG project. By working with a large data set of primary PCa (*n* = 196) we analyzed how TF binding affected somatic mutations in this disease. We found that of all the TFs, ARBS had the highest rate of mutations with a clear correlation between mutation rate and AR occupancy. Importantly, an increase in ARBS mutations was not seen in those cancers that do not express or require AR. Some ARBS were commonly affected, with ~ 2% of the patients having a mutation at these regions (Supplementary Data 1). The high frequency of mutations at specific ARBS’s suggest that these mutations may potentially provide an evolutionary advantage. Although preliminary, we demonstrated that these mutations can impact enhancer function (Fig. 5). However, additional work is needed to show that these mutations can alter gene transcription. Critically, better annotation of PCa regulatory regions is required to identify potential driver non-coding mutations.

Interestingly, the type of mutations observed at ARBS were very different than those in the remainder of the PCa genome. Specifically, we saw a high frequency of TpG- > ApG and CpG- > GpG purine transversions at both ARBS and the binding sites of HOXB13, GATA2, and KDM1A. This is not owing to overlap between the TFs, as the same type of mutations were observed at those binding sites that do not have AR co-occupancy. Previous research has demonstrated that AR activation can induce DSB by TOP2B, activation-induced cytidine deaminase or LINE-1 repeat-encoded ORF2 endonuclease^18,19^. Although there was an increase in the rate of indels at ARBS, the type of SNVs observed was not associated with DSB. In fact, compared with the remainder of the genome, ARBS had a decrease in the DSB mutational signature^1^. Our results suggest that DSBs that arise from AR-mediated transcription are efficiently repaired and do not cause a large number of SNVs within the ARBS. However, the impact of additional genotoxic stress, such as radiotherapy, on ARBS mutations requires further work. Of the AR pioneer factors, HOXB13 was found to have the highest rate of mutations. This supports recent work that demonstrated the AR cistrome of clinical PCa samples is reprogrammed from using FOXA1 to HOXB13 pioneer factors during tumourogenesis^9^. Supporting this, we observed a relatively low frequency of mutations at the binding sites of FOXA1. This raises the interesting concept that TF mutational rate can be used as a surrogate for in situ activity. Although speculative, the use of TF mutational rate may potentially help to identify clinically important pharmacological targets.

We observed a high frequency of mutations at SUZ12/EZH2-binding sites. on the type of mutations these were likely caused by 5-methylcytosine deamination. In support of this model, SUZ12/EZH2 had one of the highest rates of CpG methylation at TF-binding sites. Similar mutations were observed at the same chromosomal locations in multiple cancer types. This suggests that these regions may be prone to this particular type of damage or, more likely, that PRC2 is important in these cancers.

**Fig. 6:**
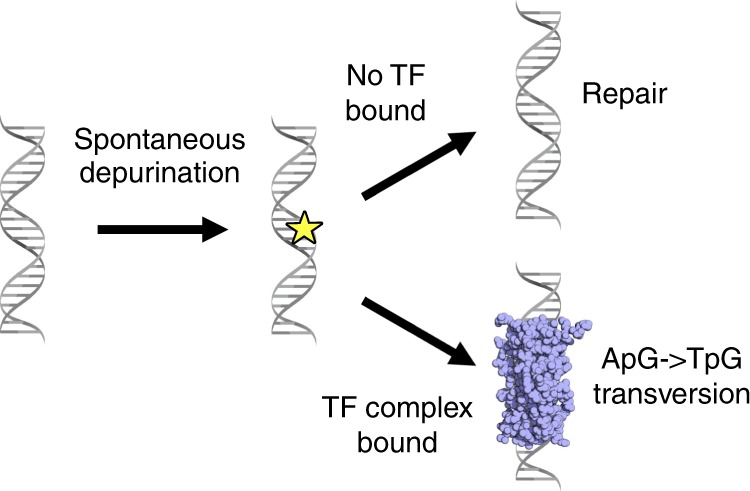
Proposed mechanism of faulty repair at abasic ARBS.

There are two potential mechanisms that could cause the observed increase in ARBS mutations. First, the AR itself may induce DNA damage when it binds to chromatin or activates gene transcription. However, mutations can also occur when repair of damaged DNA fails. In the second mechanism, a bound protein may prevent access of repair machinery to the endogenous DNA lesion. Although we cannot eliminate the first model, the increase in SNVs and remarkably similarity in mutational signature at several other TF-binding sites including KDM1A, GATA2, and HOXB13, suggests that the AR itself does not induce DNA damage. Each of these TFs bind to unique DNA sequences with different protein domains that function through disparate mechanisms. Such contrasting TFs are unlikely to induce similar damage. It is more probable that the increased rate of mutations is owing to a blockade of DNA damage repair machinery (Fig. 6). Supporting this, recent studies by Sabarinathan et al. demonstrated that in melanoma, TF binding impaired access of NER machinery^7^. By preventing the repair of UV-damaged DNA this led to a higher rate of mutations at TF-binding sites. Our results in PCa support a similar though expanded model. Specifically, we propose that TF binding prevents the repair of DNA by blocking not just NER but also BER. Several studies have demonstrated that TpG- > ApG mutations, which were observed at ARBS, arise from the failed repair of abasic sites by BER. Abasic sites frequently occur owing to spontaneous depurination at an estimated rate of 10,000 events per day per cell^37^. These endogenously damaged sites are typically repaired quickly and efficiently by APE1-mediated BER. Although abasic sites can also be repaired by NER, this is much less common^38^. In our proposed model, TF binding prevents access of BER machinery to the damaged abasic sites (Fig. 6). In support of this hypothesis, mice that have BER loss-of-function mutations accumulate endogenous DNA damage with increased rates of T- > A and C- > G purine transversions similar to that observed at ARBS^39^. Further, we also observed in vitro that the AR protein when bound to DNA reduces APE1 cleavage at abasic sites (Fig. 5). Although we did not observe a marked increase of somatic mutations at ARBS sites that contained an ARE motif, in situ an active ARBS will contain numerous co-activators, co-repressors and transcriptional machinery. This large transcriptional hub could potentially prevent access of DNA repair machinery across a larger region than just the ARE. Further, although AREs are found on ~ 1/3 of ARBS the majority of functional AR enhancers do not contain a canonical motifs^8,10^. Given the diversity of AR-driven enhancers, an ARE motif is not essential for binding or transcriptional activation. Interestingly, the deamination-associated mutations observed at SUZ12/EZH2-binding sites are also caused by a failure of BER^40^. Although NER has been shown to be impaired by TF binding to DNA, our results suggest that TF-mediated blockage may be a broader phenomenon that can impact other repair mechanisms.

Overall, this work demonstrates that somatic mutation distribution is influenced by lineage-specific TFs. We propose these mutations occur owing to faulty repair of spontaneous mutations owing to TF occupancy. These findings complement previous studies and demonstrates that the cancer cell-of-origin influences mutation patterns.

## Methods

### Expression and purification of AR-DBD

The AR-DBD (residues 556–629) in fusion with an N-terminal (His)_6_ and C-terminal–avidin tag (GLNDIFEAQKIEWHE) tag was expressed in E. Coli BL21-DE3 cells for 4 h at 20 °C. Cells were lysed in buffer containing (20 mm Tris pH 7.5, 300 mm NaCl, 5% glycerol, 20 µm ZnSO4, 4 mm dithiothreitol (DTT), 2.1 mm phenylmethylsulfonyl fluoride (PMSF)). After sonication and centrifugation, the samples were loaded onto a Ni-NTA affinity column and subsequently eluted with 300 mm imidazole. The eluted protein was then concentrated and further purified by size-exclusion chromatography equilibrated in (20 mm Tris pH 7.5, 300 mm NaCl, 5% glycerol, 20 μm ZnSO4, 1 mm DTT, 0.1 mm PMSF).

### Enzymatic cleavage of abasic sites by APE1

Complimentary oligonucleotides (5′-TACAAATAGGTTCTTGGAGTACTTTACTAGGCATGGACATAGCTGTTGACA-3′) harboring a site-specific AP site analog (tetrahydrofuran) were annealed to equal molar concentrations of the complementary strand by heating to 94 °C for 2 min and gradually cooling. Duplex DNAs (abARE, abINT, and ab3’) were then^32^P-5’-end-labeled using PNK (NEB) and standard approaches^41^. To evaluate the effects of AR binding, 0.2 pmol of the indicated duplex substrate were incubated with or without 30 pmol of AR-DBD for 30 min at room temperature. APE1 (50 pg or 1.4 fmol) was then added, and the reaction mix (final volume 10 µL) was immediately transferred to 37 °C for 10 min. Reactions were stopped and analyzed by denaturing polyacrylamide electrophoresis and phosphorimaging (Typhoon) as previously described^41^. Relative conversion rates were determined by comparing the APE1 only reaction (set as 100 for each substrate independently) with the incision efficiency [product/(product + substrate)] of the AR-DBD/APE1 reaction.

### AR enhancer activity

The genomic region of interest was cloned into the pGL4 enhancer plasmid and tested for activity in LNCaP cells that were treated with 10 nm DHT for 16 h. Cells were routinely tested for mycoplasma and the identity of LNCaP was validated by STR on 24 July 2016.

### Mutation information of ICGC patients

Whole-genome sequencing data were obtained from PCAWG release on 24 August 2016 (ref. ^28)^. For PCa only those patients with primary cancer (*n* = 196) were included in the study owing to the limited number of patients with metastatic or late-state prostate cancer. SNV and indels were previously called with three different mutation-calling algorithms (Sanger: indel = Pindel, SNV = Caveman; DKFZ: indel + SNV = Platypus; Broad: indel = Snowman, SNV = Mutect). Only those mutations which had been called by two or more callers and not found in dbSNP(v147) were used in this work. The aligned reads of two representative SNVs is shown in Supplementary Fig. 10A. The variant allelic frequency (VAF) and number of reads for ARBS SNV is shown in Supplementary Fig. 10B. Overall, the VAF of SNVs at ARBS were statistically higher than the remainder of genome (Wilcoxon rank sum test *p* *=* 0.035; Supplementary Fig. 10C). Importantly, very few called SNVs were found near called SVs suggesting that these are not mapping artifacts (Supplementary Fig. 10D). The location and frequency of the called ARBS mutations are shown in Supplementary Data 1.

### TF-binding sites

ChIPseq data were obtained from previously published studies on GEO or ENCODE (all project codes were shared under Data Availabilty section). Clinical ARBS set were generated using HOMER’s (v4.7) *mergePeaks* function (–d parameter 200)^42^. All binding sites that overlapped with UCSD blacklisted regions were removed.

Motif driven peaks were predicted by PWMtools with given positional weight matrixes obtained from JASPAR DB.

### Determination of intersecting regions

Bedtools (version 2.26.0) and bedops (version 2.4.26) were used to intersect, manipulate and filter specific regions in bed and vcf files^43^. To extend binding regions bedtools *slop* function was used. For intersection and filtration, we used bedtools *intersect* and bedops *bedmap* function.

### Comparing specific region mutation frequency with background

Bedtools *shuffle* function was used to generate randomized regions across the genome. Each bed file was randomized 1000 times to generate a null distribution. All gapped regions (UCSC gapped regions) were removed. To generate random bed files with similar base composition (ATCG) of each random region we extensively randomized the AR-binding data and then calculated base composition. We then z normalized each nucleotide type columns identify those random bed files similar to ARBS 250 bed file (as null value). The peak files that have the base composition that are in the ± 2 standard deviation (sd) range were selected.

### Mutation signature analysis

Mutation signature analysis was done using the bioconductor package SomaticSignature (version 2.12.1) with R version 3.4.0 (ref. ^44^). Mutation signature were obtained from *plotMutationSpectrum()* function with default parameters. Those TFs with < 480 mutations across all patients were not included in our analysis. This value was used as it was demonstrated to have a deciphering accuracy of > 0.95 for two mutation signature^30^.

As previously published, the *cosine()* function from the ‘lsa’ package was used to calculate the similarity between signatures obtained from SomaticSignature *motifMatrix()* function^30^

### Mutation aggregation analysis on TF-binding regions

For each of the binding regions, overlapping mutations were mapped and mutation distances to the center of the TF-binding region were calculated. For a given TF, each of the binding regions were overlapped based on their center. Mutation densities of 100 bp windows were calculated with smooth kernel density method. Calculation and visualization was conducted with ggplot2 R package.

### Methylation analysis

CpG positions were identified from a published custom made perl script (https://www.biostars.org/p/68352/#256983). DNA methylation was obtained from GEO (see Data Availability). Methylation data points with coverage less than 10 were excluded from our analysis. Those locations with a DNA methylation < 0.52 (median of LNCaP) were classified as unmethylated. Intersecting CpG of each peak was combined as a vector. Then all of the methylated and unmethylated sites were summed up to obtain single value of overall methylated rate of a TF. For given TFs, the intersection between TF and whole-genome CpG was obtained.

### Heatmap

‘pheatmap’ package (version 1.0.8) was used for drawing heatmap from CRAN package repository *pheatmap* function was used in default settings to produce heat maps based on pairwise cosine similarity values of mutation signatures.

### Statistical analysis

The distribution of mutation events limits the usage of parametric tests. For preventing biasing, we used R statistical language default *wilcox.test*() function is used for Wilcoxon rank sum test. Significance of the DNA repair and luciferase experimental assays were assessed by a two-tailed unpaired *t* test.

### Visualization

Data were visualized with *ggplot2* (version 2.2.1) and Venn diagrams were drawn in RShiny app, https://github.com/jolars/shiny-server.

### Breakpoint distance determination

Structural variation calls from Delly was obtained from ICGC repository (Release 24 August 2016). To find breakpoint locations, *sv props* script was used (https://github.com/dellytools/svprops) to process DELLY vcf files. Bedtools’ *closest* function was used to measure SNV and breakpoint distance for each patient^43^.

### Variant allele frequency and raw SNV visualization

Variant allele frequency and read number values were obtained from the ICGC consensus vcf. Extracted values were visualized by using ggplot2 package. To obtained piled-up reads of SNVs, mapped reads were downloaded with *icgc-client* following ICGC data retrieval protocol (http://docs.icgc.org/cloud/guide/#overview). For each SNV, the mapped reads (± 100 bp) were extracted and then visualized with Integrative Genome Browser (http://software.broadinstitute.org/software/igv/).

### Reporting summary

Further information on research design is available in the Nature Research Reporting Summary linked to this article.

## Supplementary information

Supplementary Information

Description of Additional Supplementary Files

Supplementary Data 1

Reporting Summary

## Data Availability

ChIPseq data were obtained for the following published work: FOXA1 (GSM1410788), CHD1 (GSM1573653), CTBP1 (GSM1410762), CTBP2 (GSM1410763), CTCF (GSM1006887), ETV1 (GSM1145322), EZH2 (GSM969570), GATA2 (GSM941194), GRLH2 (GSM2122802), HOXB13 (GSM1716764), MRE11A (GSM1543776), POLR2A (GSM1415124), RUNX1 (GSM1527840), SUZ12 (GSM969572), TCF7L2 (GSM1249449), TET2 (GSM1613322), TOP1 (GSM1543792), WDR5 (GSM1333369), KDM1A (GSM1279769), EP300 (GSM686943), MED12 (GSM686945), AR (GSE83860), H3K9ME3 (GSM353610), H3K4ME1 (GSM1410780), H3K4ME3(ENCODE: ENCFF401MDR), H3K27AC (GSM1249448), H3K36ME3 (GSM875814). Clinical ARBS were identified from AR ChIPseq of 13 tumor and 7 normal human tissue samples (GSE70079). WGS data were obtained from PCAWG release on 24 August 2016 (ref. ^28^). DNA methylation was quantified from whole genome bisulfide sequencing from LNCaP cells (GSE86832).
