# Supplementary material for: Androgen receptor-binding sites are highly mutated in prostate cancer
Source: Nat Commun. 2020 Feb 11;11:832. doi: 10.1038/s41467-020-14644-y (PMC7012874; doi:10.1038/s41467-020-14644-y)
Supplement: Supplementary file 1 — Supplementary Information [file 41467_2020_14644_MOESM1_ESM.pdf]

Supplementary Figure 1

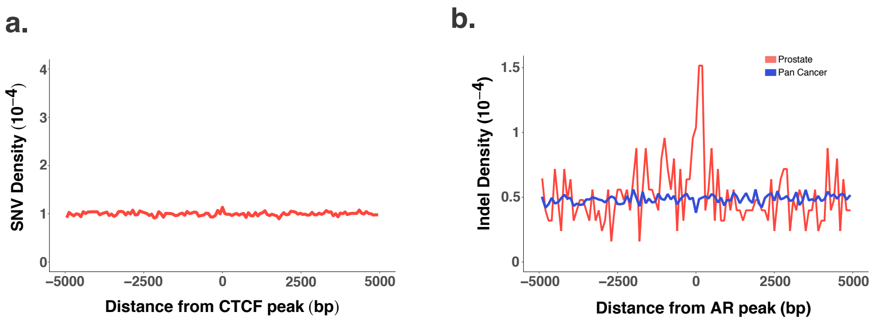

Supplementary Figure 1: (*A*) SNV Density at CTCF binding sites in PCa. Unlike previous work in melanoma and colorectal cancer, CTCF binding sites have no increase of SNVs in in PCa. (*B*) Indel density in PCa and all other cancers (pan cancer). The rate of indels at ARBS was increased in PCa but not other cancers types.

Supplementary Figure 2

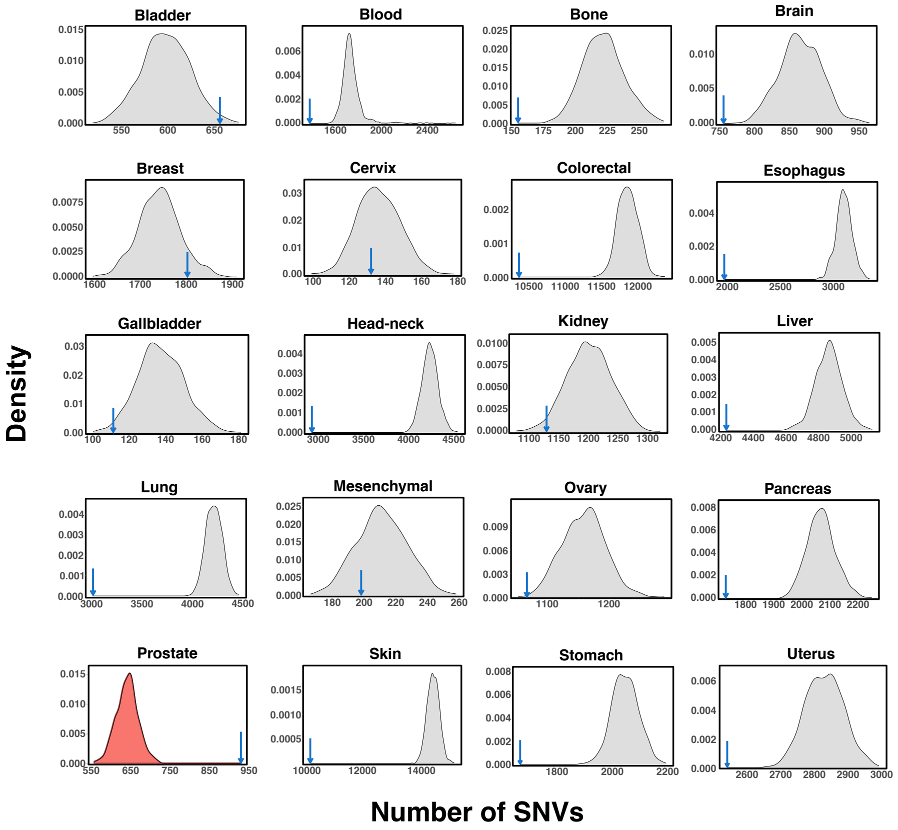

Supplementary Figure 2: Randomization of ARBS mutations in other cancers. The rate of SNVs at ARBS (blue arrow) was compared to a randomized region (grey) in all cancers with greater than 500 total SNVs. Only PCa (red) had a higher rate of mutations at ARBS than the null distribution.

Supplementary Figure 3

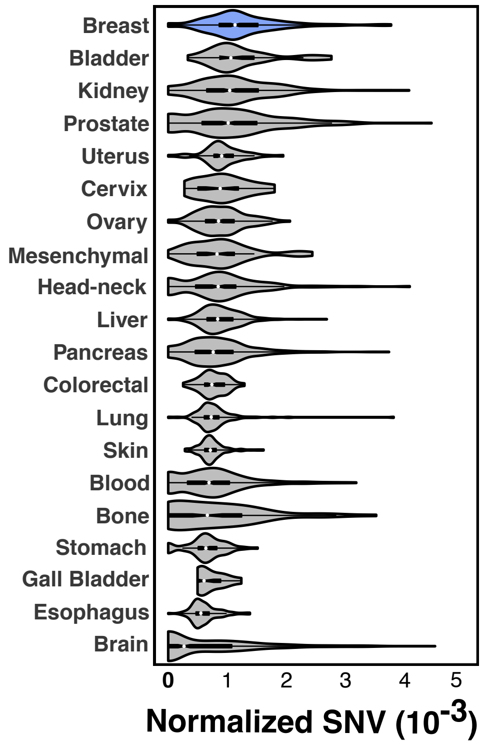

Supplementary Figure 3: Estrogen Receptor has the highest rate of mutations in breast cancer. The normalized rate of SNVs at ERBS was compared in multiple cancers. Breast cancer has found to have the highest rate of SNVs at these binding sites.

Supplementary Figure 4

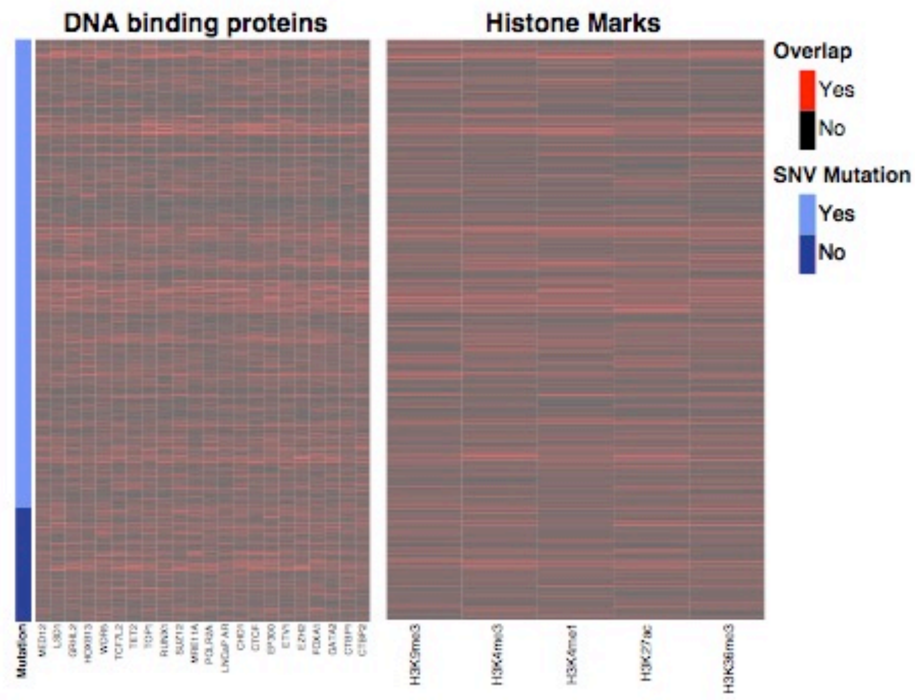

Supplementary Figure 4: ARBS mutations were clustered with all other histone marks and TFs. No correlation was observed between the ARBS SNV and specific epigenetic marks or TF binding.

Supplementary Figure 5

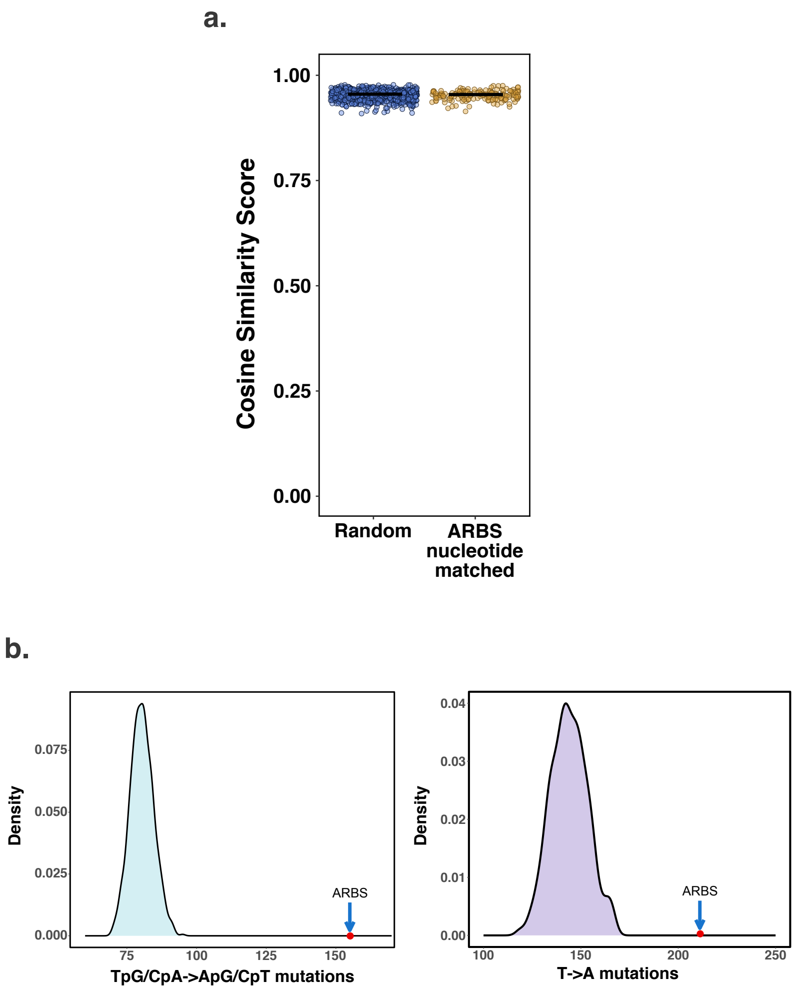

Supplementary Figure 5: The mutational signature at ARBS is not due to nucleotide composition. (*A*) The mutational signature of PCa whole genome was compared against randomized regions (n=1000) that the same size as ARBS (~100k Kb). Randomized regions that had a matched nucleotide composition to the ARBS were subsetted (yellow). The mutational signature of the whole genome was extremely similar to the smaller randomized regions with a median cosine similarity of 0.953. (*B*) ARBS were found to have a higher frequency of all T->A or TpG->ApG transversion than randomized regions with a matched nucleotide composition.

Supplementary Figure 6

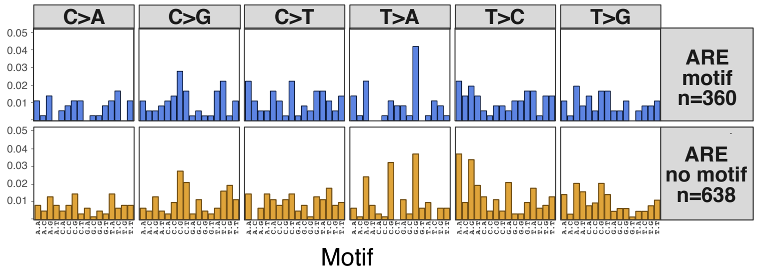

Supplementary Figure 6: The frequency and type of ARBS mutations were not impacted by the presence of a canonical Androgen Response Element (ARE) motif.

Supplementary Figure 7

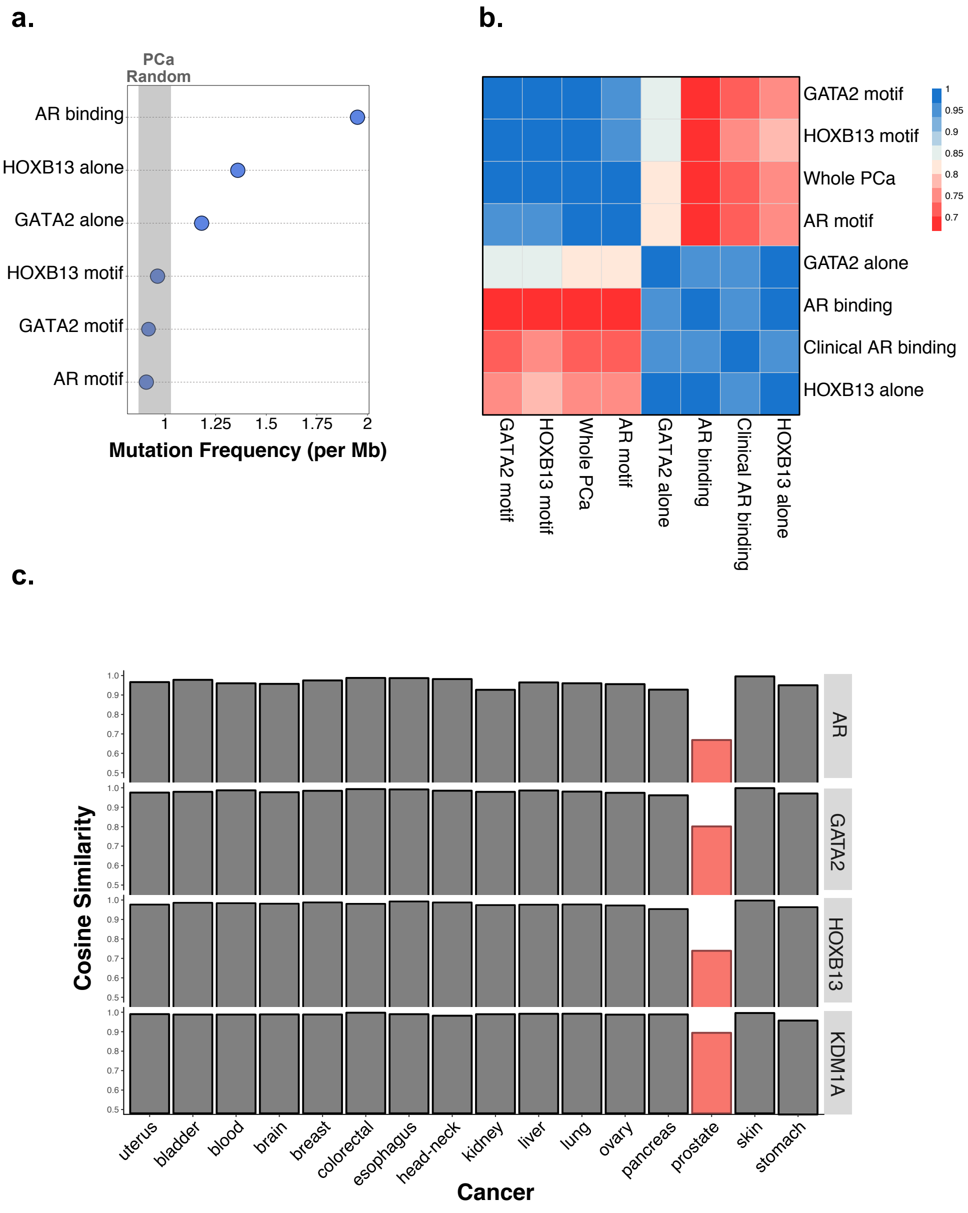

Supplementary Figure 7: TF binding is required for an increase in SNV and altered mutation signature. (*A*) Mutation frequency was calculated at those sites that had a specific TF motif but no protein to those TF binding sites. Those regions which had the motif but no protein did not have a greater rate of mutations than randomize regiosn in the genome (grey)(*B*) Mutational signatures at motif containing and TF binding sites were calculated and analyzed using a cosine similarity measure. Motif signatures and ChIP-seq signatures appeared in two distinct clusters with the regions containing the motif being very similar to the whole PCa. (*C*) The cosine similarity of somatic mutations that occur at specific TF binding sites and the remainder of the cancer genome were calculated for all cancer types. Only PCa had a different mutational signature at these TF binding sites.

Supplementary Figure 8

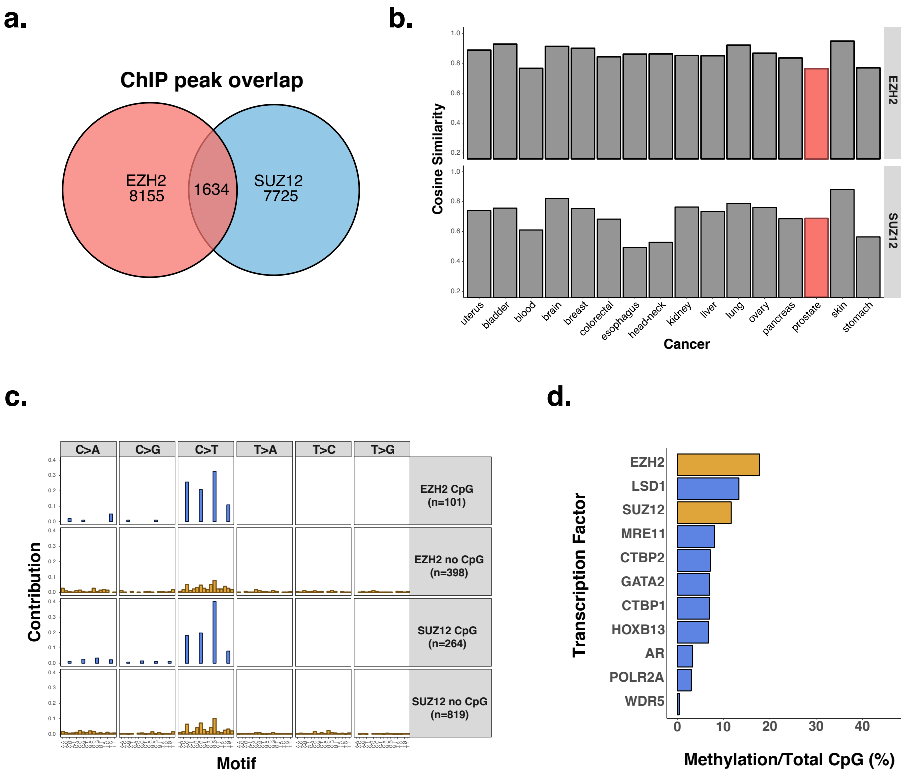

Supplementary Figure 8: mutation signature of EZH2 and SUZ12 is correlated with their methylation rates. *A*) EZH2 and SUZ12 ChIP-seq peaks shows a relatively low numbers of overlapping regions in LCNaP cells. (*B*) The mutation signature at SUZ12 and EZH2 binding sites is very different than the remainder of the genome in multiple cancer. (*C*) EZH2/SUZ12 mutation signatures were calculated at regions with CpG and non-CpG. C->T mutations were significantly enriched at CpG regions. (*D*) Overall methylation rate at TF binding sites was determined from whole genome bisulfite sequencing.

Supplementary Figure 9

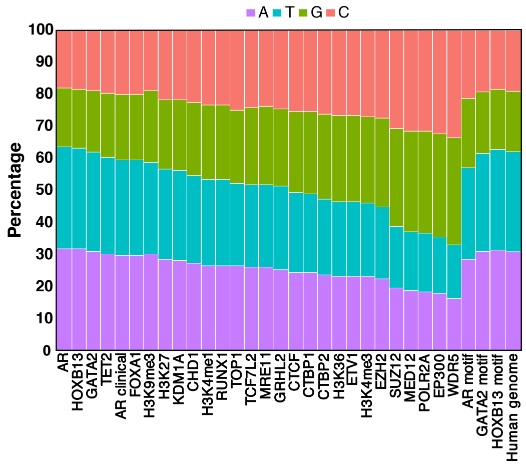

Supplementary Figure 9: ATCG composition of al TF binding sites and histone marks. The GC and AT nucleotide composition was calculated at all TF binding sites, histone marks and regions that contain specific TF motifs.

Supplementary Figure 10

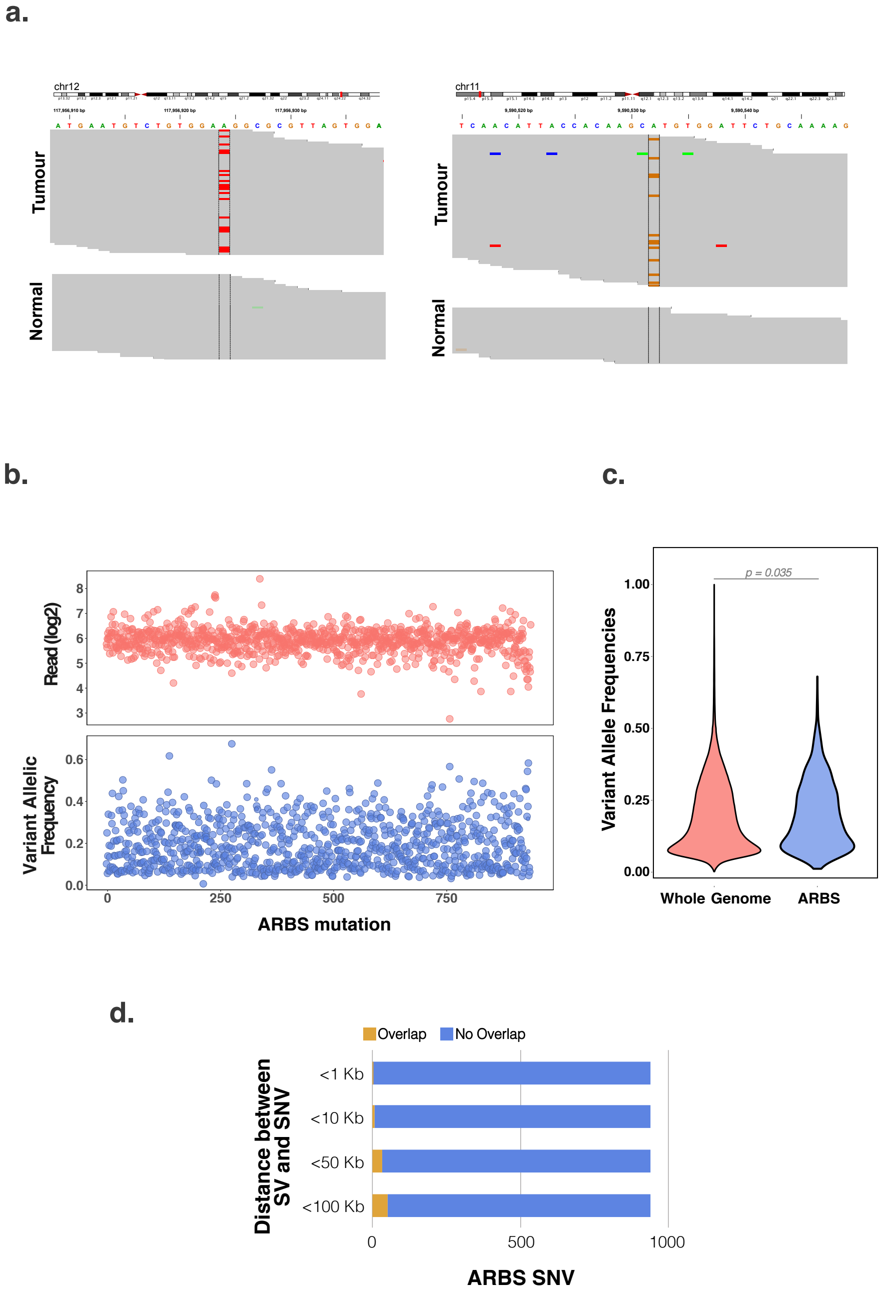

Supplementary Figure 10: (*A*) Raw sequencing reads of tumour and normal at called SNVs in two patients were visualised. Mutated basepairs are coloured while those that match are grey. (*B*) Read depth and variant allele frequency of all ARBS mutations in PCa investigation show no relation between read depth and variant allele frequency (Pearson correlation = -.025). (*C*) SNV mutations at ARBS have a significantly higher variant allelic frequency than remainder of genome (Wilcoxon test p = .03515). (*D*) The called ARBS SNVs are distant to SVs. The number of SNVs found within fixed distance to the SV are shown (<1 Kb, <10 Kb, <50 Kb, <100 Kb).
