# Supplementary material for: Androgen receptor-binding sites are highly mutated in prostate cancer
Source: Nat Commun. 2020 Feb 11;11:832. doi: 10.1038/s41467-020-14644-y (PMC7012874; doi:10.1038/s41467-020-14644-y)
Supplement: Supplementary file 2 — Description of Additional Supplementary Files [file 41467_2020_14644_MOESM2_ESM.pdf]

Description of Additional Supplementary Files

File Name: Supplementary Data 1

Description: List of mutations which overlapped with ARBS
