# Supplementary material for: Androgen receptor-binding sites are highly mutated in prostate cancer
Source: Nat Commun. 2020 Feb 11;11:832. doi: 10.1038/s41467-020-14644-y (PMC7012874; doi:10.1038/s41467-020-14644-y)
Supplement: Supplementary file 4 — Reporting Summary [file 41467_2020_14644_MOESM4_ESM.pdf]

nature research

Corresponding author(s): Nathan A. Lack
Last updated by author(s): Jan 9, 2020

Reporting Summary

Nature Research wishes to improve the reproducibility of the work that we publish. This form provides structure for consistency and transparency in reporting. For further information on Nature Research policies, see Authors & Referees and the Editorial Policy Checklist.

Statistics

For all statistical analyses, confirm that the following items are present in the figure legend, table legend, main text, or Methods section.

n/a	Confirmed	
☐	☒	The exact sample size (*n*) for each experimental group/condition, given as a discrete number and unit of measurement
☐	☒	A statement on whether measurements were taken from distinct samples or whether the same sample was measured repeatedly
☐	☒	The statistical test(s) used AND whether they are one- or two-sided<br>*Only common tests should be described solely by name; describe more complex techniques in the Methods section.*
☐	☒	A description of all covariates tested
☐	☒	A description of any assumptions or corrections, such as tests of normality and adjustment for multiple comparisons
☐	☒	A full description of the statistical parameters including central tendency (e.g. means) or other basic estimates (e.g. regression coefficient) AND variation (e.g. standard deviation) or associated estimates of uncertainty (e.g. confidence intervals)
☐	☒	For null hypothesis testing, the test statistic (e.g. *F*, *t*, *r*) with confidence intervals, effect sizes, degrees of freedom and *P* value noted<br>*Give P values as exact values whenever suitable.*
☒	☐	For Bayesian analysis, information on the choice of priors and Markov chain Monte Carlo settings
☒	☐	For hierarchical and complex designs, identification of the appropriate level for tests and full reporting of outcomes
☒	☐	Estimates of effect sizes (e.g. Cohen's *d*, Pearson's *r*), indicating how they were calculated

Our web collection on statistics for biologists contains articles on many of the points above.

Software and code

Policy information about availability of computer code

Data collection	We shared the more detailed information about in the methods section of our manuscript.
Data analysis	We implemented set of tools such as bedtools(2.26.0), bedops(2.4.26). In addition to this we used R (3.4.0) with following packages SomaticSignatures(2.12.1), pheatmap (1.0.8) and ggplot2(2.2.1).

For manuscripts utilizing custom algorithms or software that are central to the research but not yet described in published literature, software must be made available to editors/reviewers. We strongly encourage code deposition in a community repository (e.g. GitHub). See the Nature Research guidelines for submitting code & software for further information.

Data

Policy information about availability of data

All manuscripts must include a data availability statement. This statement should provide the following information, where applicable:
- Accession codes, unique identifiers, or web links for publicly available datasets
- A list of figures that have associated raw data
- A description of any restrictions on data availability

All ChIP-seq and mutation data is described in the methods section of our manuscript. Briefly this includes, ChIP-seq data from the following published work: FOXA1(GSM1410788), CHD1(GSM1573653), CTBP1(GSM1410762), CTBP2(GSM1410763), CTCF(GSM1006887), ETV1(GSM1145322), EZH2(GSM969570), GATA2(GSM941194), GRLH2(GSM2122802), HOXB13(GSM1716764), MRE11A(GSM1543776), POLR2A(GSM1415124), RUNX1(GSM1527840), SUZ12(GSM969572), TCF7L2(GSM1249449), TET2(GSM1613322), TOP1(GSM1543792), WDR5(GSM1333369), KDM1A(GSM1279769), EP300(GSM686943), MED12(GSM686945), AR(GSE83860), H3K9ME3(GSM353610), H3K4ME1(GSM1410780), H3K4ME3(ENCODE: ENCFF401MDR), H3K27AC(GSM1249448), H3K36ME3(GSM875814). Clinical ARBS were identified from AR ChIP-seq of 13 tumour and 7 normal human tissue samples (GSE70079). Whole genome sequencing data was obtained from Pan Cancer Analysis of Whole Genomes (PCAWG) release on August 24, 2016 (https://dcc.icgc.org/)

Field-specific reporting

Please select the one below that is the best fit for your research. If you are not sure, read the appropriate sections before making your selection.

☒ Life sciences ☐ Behavioural & social sciences ☐ Ecological, evolutionary & environmental sciences

For a reference copy of the document with all sections, see nature.com/documents/nr-reporting-summary-flat.pdf

Life sciences study design

All studies must disclose on these points even when the disclosure is negative.

Sample size	WGS was analyzed from 196 primary PCa tumours and 2576 non-PCa cancers.
Data exclusions	WGS from advanced or metastatic PCa samples were removed from the ICGC cohort due to the low number of patients
Replication	For all experimental validation studies a minimum of three biological replicas were used.
Randomization	No randomization was included as this was a descriptive publication.
Blinding	No blinding was done as this was a descriptive publication.

Reporting for specific materials, systems and methods

We require information from authors about some types of materials, experimental systems and methods used in many studies. Here, indicate whether each material, system or method listed is relevant to your study. If you are not sure if a list item applies to your research, read the appropriate section before selecting a response.

Materials & experimental systems

n/a	Involved in the study	
☒	☐	Antibodies
☐	☒	Eukaryotic cell lines
☒	☐	Palaeontology
☒	☐	Animals and other organisms
☒	☐	Human research participants
☒	☐	Clinical data

Methods

n/a	Involved in the study	
☒	☐	ChIP-seq
☒	☐	Flow cytometry
☒	☐	MRI-based neuroimaging

Eukaryotic cell lines

Policy information about cell lines

Cell line source(s)	ATCC
Authentication	STR profiling
Mycoplasma contamination	Mycoplasma was tested for bi-weekly
Commonly misidentified lines (See ICLAC register)	N/A
